# Zirconia/dental pulp stem cell composite scaffolds repair osteogenic defect via regulating macrophages

**DOI:** 10.3389/fbioe.2025.1632000

**Published:** 2025-08-29

**Authors:** Yingying Wu, Maodian He, Cuimei Li, Weihua Shang, Jiale Hu, Bingyao Liu

**Affiliations:** Department of Stomatology, Taikang Xianlin Drum Tower Hospital, Affiliated Hospital of Medical School, Nanjing University, Nanjing, Jiangsu, China

**Keywords:** dental pulp stem cells, bone regeneration, macrophage polarization, MAPK signaling, zirconia

## Abstract

**Background:**

Dental pulp stem cells (DPSCs) possess multilineage differentiation potential and immunomodulatory properties, making them promising candidates for bone regeneration. In this study, the regenerative potential of DPSCs combined with nitrogen-doped reduced graphene oxide/zirconia (N-Rgo/ZrO_2_) composite scaffolds was investigated *in vitro* and in a rat jaw injury model.

**Methods:**

The primary human DPSCs were characterized by flow cytometry (CD90/CD29/CD45) and trilineage differentiation assays. *In vitro* effects on macrophage polarization (IL-6, TNF-α, CD206, Arg1, iNOS, IL-10) and MAPK signaling (p-ERK1/2, p-p38) were analyzed using qRT-PCR and Western blot. A rat bone defect model was established to evaluate mandibular bone regeneration through H&E staining, Masson’s trichrome, and molecular analysis of osteogenic markers (RUNX2, ALP, Osterix, OPN, OCN) and pathway activation.

**Results:**

DPSCs exhibited characteristic mesenchymal markers (CD90^+^: 90.16% ± 1.00%; CD29^+^: 99.27% ± 0.11%) and multilineage differentiation capacity. N-Rgo/ZrO2+DPSCs synergistically upregulated M2 macrophage markers (CD206, Arg1, IL-10) and pro-osteogenic cytokines (TNF-α) while suppressing IL-6. This combination potently activated p-ERK1/2 and p-p38 MAPK, exceeding single-treatment effects. *In vivo*, cotreatment restored trabecular architecture (collagen fiber density), improved rat pathological conditions, and significantly elevated osteogenic markers (RUNX2, ALP, Osterix, OPN and OCN) compared with monotherapies.

**Conclusion:**

The N-Rgo/ZrO2+DPSCs composite promotes bone regeneration through dual mechanisms: (1) immunomodulation through M2 macrophage polarization and MAPK pathway activation and (2) direct osteogenic differentiation. This strategy demonstrates superior efficacy to individual components, offering a novel combinatorial approach for maxillofacial bone repair.

## 1 Introduction

Bone regeneration in maxillofacial defects remains a significant clinical challenge, particularly in cases of trauma, osteoporosis, or post-resection rehabilitation (Donos et al., 2023). Although autografts are the “gold standard,” limitations, such as donor-site morbidity and insufficient supply, have led researchers to explore alternative strategies, including stem-cell-based tissue engineering (Tsuk et al., 2022). Among stem cell sources, dental pulp stem cells (DPSCs) have emerged as promising candidates owing to their high proliferative capacity, multilineage differentiation potential (including osteogenesis), and unique immunomodulatory properties (Sui et al., 2020; Calabrese et al., 2022). However, the efficacy of DPSCs in bone repair depends highly on their microenvironment, particularly their interactions with biomaterials and host immune cells (Han et al., 2021).

DPSCs are mesenchymal stem cells (MSCs) derived from the neural crest that exhibit superior proliferative capacity, multilineage differentiation potential, and immunomodulatory properties compared with other MSC sources (Ercal et al., 2018). DPSCs express characteristic MSC markers (e.g., CD90 and CD29) while lacking hematopoietic markers (e.g., CD45), confirming their stromal origin (Gallorini et al., 2024). Their osteogenic potential is well documented, with robust expression of bone-related markers (RUNX2, ALP, and OCN) under inductive conditions (Kor et al., 2022). DPSCs secrete paracrine factors (e.g., VEGF and BMP-2) that promote angiogenesis and tissue repair, and their ability to polarize macrophages toward an M2 phenotype enhances regenerative microenvironments (Yang et al., 2023). These properties, combined with their minimally invasive isolation from extracted teeth, make DPSCs an attractive candidate for bone tissue engineering, particularly in craniofacial applications (Kwack and Lee, 2022).

Macrophages play a pivotal role in bone regeneration by modulating inflammation and tissue remodeling through polarization into pro-inflammatory (M1) or anti-inflammatory/pro-regenerative (M2) phenotypes (Boutilier and Elsawa, 2021). Emerging evidence suggests that DPSCs can induce M2 macrophage polarization, thereby creating a pro-osteogenic milieu (Anderson et al., 2022). However, the molecular mechanisms underlying this crosstalk—especially the involvement of important pathways, such as MAPK (ERK1/2, p38)—are poorly characterized (Wang et al., 2024). Furthermore, the physicochemical properties of a scaffold can significantly influence macrophage behavior and stem cell differentiation (Sadtler et al., 2017). Zirconia (ZrO_2_)-based composites have gained prominence in bone tissue engineering because of their exceptional mechanical strength, biocompatibility, and osteoconductivity, which are critical for load-bearing maxillofacial applications (Ji et al., 2022). However, pure ZrO_2_ lacks sufficient bioactivity to support cell adhesion and osteogenic differentiation fully. To address this, nitrogen-doped reduced graphene oxide (N-Rgo) has been incorporated into ZrO_2_ matrices to form functional nanocomposites. N-Rgo enhances scaffold performance by (1) improving electrical conductivity, which promotes cellular communication and osteogenic signaling (Yadav et al., 2022; Tsuk et al., 2022) providing a nanotopographical surface that mimics the bone extracellular matrix (ECM), thereby facilitating DPSC attachment and proliferation, and (Sui et al., 2020) offering tunable hydrophilicity for optimal protein adsorption and cytokine retention (Luis-Sunga et al., 2024). In addition, the high surface area and chemical stability of N-Rgo make possible the controlled release of bioactive molecules, further augmenting immunomodulation and osteogenesis (Zhang and Wu, 2022). However, it remains unclear whether such composites can synergize with DPSCs to amplify immunomodulation and osteogenesis.

Current limitations in this field include (1) insufficient knowledge of DPSC–macrophage–scaffold tripartite interactions (2), lack of combinatorial strategies to simultaneously optimize immunomodulation and osteogenesis, and (3) limited *in vivo* validation of these mechanisms in clinically relevant models (e.g., osteoporotic bone defects) [12,13]. To address these gaps, in a previous study, N-Rgo/ZrO_2_ composite scaffolds were successfully prepared, and the combination of N-Rgo/ZrO_2_ composite scaffolds and DPSCs regulated the migration, polarization, and glycolysis of macrophages *in vitro* (Liu et al., 2024). Therefore, in this study, the regenerative potential of N-Rgo/ZrO_2_ composite scaffolds loaded with DPSCs was investigated further both *in vitro* and in a rat model of mandibular defects. It was hypothesized that the composite scaffolds would enhance bone repair by two mechanisms (1): promoting M2 macrophage polarization to suppress chronic inflammation and (2) activating MAPK signaling to promote osteogenic differentiation.

## 2 Materials and methods

### 2.1 Isolation and identification of DPSCs

The primary human dental pulp stem cells (hDPSCs) were obtained from Fuheng Biology (Shanghai, China, https://www.fudancell.com/sys-pd/4315.html), and were cultured in α-Minimum Essential Medium (Beyotime, Shanghai, China) supplemented with 10% fetal bovine serum (FBS, Beyotime, Shanghai, China), 100 U/mL penicillin, and 100 μg/mL streptomycin in a 37 °C, 5% CO_2_ incubator with medium renewal every two to 3 days. Fourth-passage hDPSCs in the logarithmic growth phase were washed twice with PBS, enzymatically detached, centrifuged, and resuspended in PBS containing 2% FBS. After centrifugation, cell suspensions (100 μL) were aliquoted into flow cytometry tubes and incubated with CD45-FITC (cat. no. 368507, BioLegend, San Diego, CA, USA), CD29-FITC (cat. no. MA290030; Antigenix, NJ, USA), and CD90-FITC (cat. no. 328107; BioLegend) antibodies (with isotype controls) in the dark at 4 °C for 45 min. Following two PBS washes, surface marker expression was analyzed using a flow cytometer.

The osteogenic, chondrogenic, and adipogenic differentiations were performed using Alizarin Red S, Alcian Blue, and Oil Red O, respectively. For osteogenesis, hDPSCs were cultured in osteogenic induction medium (supplemented with 0.01-μM dexamethasone and 1.8-mM KH2PO4), and the medium was replaced every 3 days. After being incubated for 21 days, to observe mineralized deposits, the cells were fixed with 4% paraformaldehyde and then stained with 2% Alizarin Red S solution (Servicebio, Wuhan, China) at room temperature for 30 min. For chondrogenesis, hDPSCs were cultured in chondrogenic induction medium (supplemented with 1-mM sodium pyruvate, 0.1-mM dexamethasone, 1× insulin-transferrin-selenium-X, 10-ng/mL TGF-β, and 0.1-mM L-ascorbic acid 2-phosphate) for 21 days. After being fixed with 4% paraformaldehyde, the hDPSCs were stained with Alcian Blue (1% in 3% acetic acid, Servicebio) for 40 min, 0.1-M hydrochloric acid was used to remove the unstained areas, and then they were neutralized with water. For adipogenesis, hDPSCs were cultured in adipogenic induction medium (supplemented with 10-μg/mL insulin, 0.5-μM hydrocortisone, 500-μM 3-isobutyl-1-methylxanthine, and 60-μM indomethacin) for 21 days. After being fixed and washed, the hDPSCs were stained with Oil Red O (0.3% in 60% isopropanol, Servicebio) for 30 min in the dark. After staining, the cell images were observed and acquired under a light microscope (Eclipse TS100, Nikon Corporation, Tokyo, Japan) for lineage-specific matrix evaluation.

### 2.2 Preparation of experimental materials

Graphene oxide (GO) was synthesized using the modified Hummers’ method. Nitrogen doping was achieved by thermal annealing (800 °C, 2 h) under a NH_3_ atmosphere. Zirconium oxychloride (ZrOCl_2_·8H_2_O) was hydrolyzed in N-RGO suspension (1 mg/mL) under ultrasonication. The mixture was hydrothermally treated (180 °C, 12 h), washed, and freeze-dried to obtain graphene oxide/zirconia (N-Rgo/ZrO_2_) porous scaffolds. Then, DPSCs were seeded on N-RGO/ZrO_2_ scaffolds as previously reported (Liu et al., 2024). Briefly, N-RGO/ZrO2 scaffolds (Φ5 × 2 mm) were ultraviolet (UV)-sterilized and prewetted in osteogenic medium (24 h). Passage 3 DPSCs (1 × 106 cells/scaffold) were seeded dropwise and cultured for 7 days (37 °C, 5% CO_2_). After 7 days of cultivation, zirconia combined with dental pulp stem cell (N-Rgo/ZrO_2_+DPSCs) composite scaffolds were obtained.

### 2.3 Cell culture and grouping

A coculture system of N-Rgo/ZrO_2_ composite scaffolds and DPSCs (Cyagen Biosciences, Shanghai, China) was established using Transwell technology (Liu et al., 2024). N-Rgo/ZrO_2_, DPSCs, or N-Rgo/ZrO2+DPSCs composite scaffolds were placed in the lower chamber, while Thp-1 macrophages (Chinese Academy of Sciences Cell Bank, Shanghai, China) were cultured in the upper chamber. The experimental groups were as follows: control, N-Rgo/ZrO_2_, DPSCs, and N-Rgo/ZrO_2_+DPSCs group. Each group of cells was cultured in a cell culture incubator at 37 °C and 5% CO_2_ for 48 h, and the cells cultured for 48 h were harvested for subsequent experiments (Liu et al., 2024).

### 2.4 Animals and treatments

Adult female Wistar rats (approximately 200 g, specific pathogen-free grade) were obtained from Shanghai Slack Laboratory Animal Co., Ltd. (Shanghai, China) and acclimatized for 1 week under standard laboratory conditions with *ad libitum* access to rodent chow, maintained at 22 °C–25 °C and 20%–25% relative humidity. Following anesthesia, the left submandibular region was aseptically prepared, and a parallel incision along the inferior mandibular border was made to expose the skin, subcutaneous tissue, and musculoperiosteal layers sequentially via blunt dissection. A full-thickness circular bone defect was created in the mandibular body by using a dental round bur. Defects were implanted with designated scaffold materials according to the experimental groups: control, model, DPSCs, N-Rgo/ZrO_2_, and N-Rgo/ZrO_2_+DPSCs (6 rats/group). Layered closure of the periosteum, muscle, and skin was performed. Postoperative penicillin (10,000 IU/kg, intramuscular) was administered daily for 3 days to prevent infection. At 8 weeks after modeling, rats were euthanized, and mandibular specimens were collected for fixation in 4% paraformaldehyde or storage at −80 °C for subsequent analysis. All the animal experiments were approved by the Animal Care and Ethics Committee of Taikang Xianlin Drum Tower Hospital, Affiliated Hospital of Medical School, Nanjing University (approval no. 2019JLHSKJDWLS-001).

### 2.5 Hematoxylin and eosin (HE) staining

Tissue sections (4 μm) fixed in 4% paraformaldehyde and paraffin embedded were deparaffinized, rehydrated, stained with hematoxylin (5 min), differentiated in 1% acid ethanol, blued in Scott’s tap water substitute, counterstained with eosin Y (2 min), dehydrated through graded alcohols, cleared in xylene, and mounted with resinous medium for microscopic evaluation of nuclear (blue-purple) and cytoplasmic/extracellular matrix (pink) morphology under light microscopy.

### 2.6 Masson’s trichrome staining

Tissues were paraffin embedded and cut into 4-μm sections. After deparaffinization and rehydration, the samples underwent Masson’s trichrome staining using Weigert’s hematoxylin (5 min), Biebrich scarlet-acid fuchsin (10 min), and aniline blue (5 min), with 1% phosphomolybdic acid differentiation (5 min) prior to dehydration and mounting. Collagen fibers were stained blue, cytoplasm/muscle red, and nuclei dark blue/black, making quantitative assessment of fibrosis or collagen distribution possible using light microscopy (Nikon Eclipse E100).

### 2.7 Real-time quantitative PCR (RT-qPCR)

The RNA was isolated from cells or tissue samples (after grinding) using TRIzol reagent (Beyotime, Shanghai, China), and the quality and concentration of the extracted RNA were assessed using a microplate reader (Thermo Fisher Scientific). Subsequently, 1 μg of RNA was reverse-transcribed into cDNA using the PrimeScript II 1st Strand cDNA Synthesis Kit (Takara, Japan) based on the manufacturer’s instructions. RT-qPCR was conducted using SYBR Green Master Mix (Bio-Rad, USA) on an ABI 7500 thermocycler (Applied Biosystems, CA, USA). The reaction conditions were 95 °C for 10 min, 95 °C for 15 s, 60 °C for 1 min, and amplification for 35 cycles. Gene expression levels were quantified using the 2^−ΔΔCt^ method, with primer sequences detailed in Table 1.

**TABLE 1:** Sequences of all primers.

Primer	Sequences (5′-3′)
IL-6-hF	CCT​GAA​CCT​TCC​AAA​GAT​GGC
IL-6-hR	TTC​ACC​AGG​CAA​GTC​TCC​TCA
TNF-α-hF	GAG​GCC​AAG​CCC​TGG​TAT​G
TNF-α-hR	CGG​GCC​GAT​TGA​TCT​CAG​C
iNOS-hF	TTC​AGT​ATC​ACA​ACC​TCA​GCA​AG
iNOS-hR	TGG​ACC​TGC​AAG​TTA​AAA​TCC​C
CD206-hF	TCC​GGG​TGC​TGT​TCT​CCT​A
CD206-hR	CCA​GTC​TGT​TTT​TGA​TGG​CAC​T
IL-10-hF	GAC​TTT​AAG​GGT​TAC​CTG​GGT​TG
IL-10-hR	TCA​CAT​GCG​CCT​TGA​TGT​CTG
Arg1-hF	GTG​GAA​ACT​TGC​ATG​GAC​AAC
Arg1-hR	AAT​CCT​GGC​ACA​TCG​GGA​ATC
GAPDH-hF	TGA​CAA​CTT​TGG​TAT​CGT​GGA​AGG
GAPDH-hR	AGG​CAG​GGA​TGA​TGT​TCT​GGA​GAG
IL-6-rF	AAG​AAA​GAC​AAA​GCC​AGA​GTC
IL-6-rR	CAC​AAA​CTG​ATA​TGC​TTA​GGC
TNF-α-rF	TCA​GCC​TCT​TCT​CAT​TCC​TGC
TNF-α-rR	TTG​GTG​GTT​TGC​TAC​GAC​GTG
iNOS-rF	ATC​CCG​AAA​CGC​TAC​ACT​T
iNOS-rR	CGGCTGGACTTCTCACTC
IL-10-rF	AGA​AGG​ACC​AGC​TGG​ACA​ACA​T
IL-10-rR	CAA​GTA​ACC​CTT​AAA​GTC​CTG​CAG​TA
Arg1-rF	CAGTGGCGTTGACCTTGT
Arg1-rR	TGGTTCTGTTCGGTTTGC
GAPDH-rF	AGA​CAG​CCG​CAT​CTT​CTT​GT
GAPDH-rR	CTT​GCC​GTG​GGT​AGA​GTC​AT

### 2.8 Western blot

Total proteins were extracted from cells or tissues (after grinding) using RIPA lysis buffer (Beyotime), followed by quantification using a bicinchoninic acid (BCA) assay kit (Beyotime). The isolated protein samples (20 μg) were then separated using 10% SDS-PAGE and transferred to PVDF membranes (Millipore, MA, USA). The membranes were incubated with specific primary antibodies. At the end of the incubation, the bound antibodies were detected using a colorimetric reaction to determine the expression of the target proteins. The following antibodies were used in this study: anti-RUNX2 (cat. no. 20700-1-AP, Proteintech, Wuhan, China, 1:1000), anti-ALP (cat. no. bsm-52252R, Bioss, Beijing, China, 1:1000), anti-Osterix (cat. no. bs-25532R, Bioss, 1:1000), anti-OPN (cat. no. 30200-1-AP, Proteintech, 1:1000), anti-OCN (cat. no. 16157-1-AP, Proteintech, 1:1000), anti-p38 (cat. no. 14064-1-AP, Proteintech, 1:1000), anti-p-p38 (cat. no. 4511, CST, Boston, USA, 1:1000), anti-ERK1/2 (cat. no. 11257-1-AP, Proteintech, 1:1000), anti-p-ERK1/2 (cat. no. 4370, CST, 1:1000), and anti-GAPDH (cat. no. 60004-1-Ig, Proteintech, 1:1000). After washing with TBST solution (Takara, Shiga, Japan), the membranes were incubated with an HRP-conjugated secondary antibody (cat. no. 111–035-003 or cat. no. 115–035-003, Jackson ImmunoResearch, Lancaster, USA, 1:5000) for 1 h. The signal was developed using an enhanced chemiluminescence immunoblotting detection reagent (Thermo Fisher Scientific, MA, USA).

### 2.9 Statistical analysis

All data were analyzed using GraphPad Prism 8.4.0 software, and the data were expressed as mean ± standard deviation (SD). When comparing two groups, the t-test was used. However, to assess disparities among more than two groups, one-way analysis of variance (ANOVA) was used, followed by Tukey’s *post hoc* test. Statistical significance was defined as P < 0.05.

## 3 Results

### 3.1 Identification and differentiative capacity of DPSCs

**FIGURE 1:**
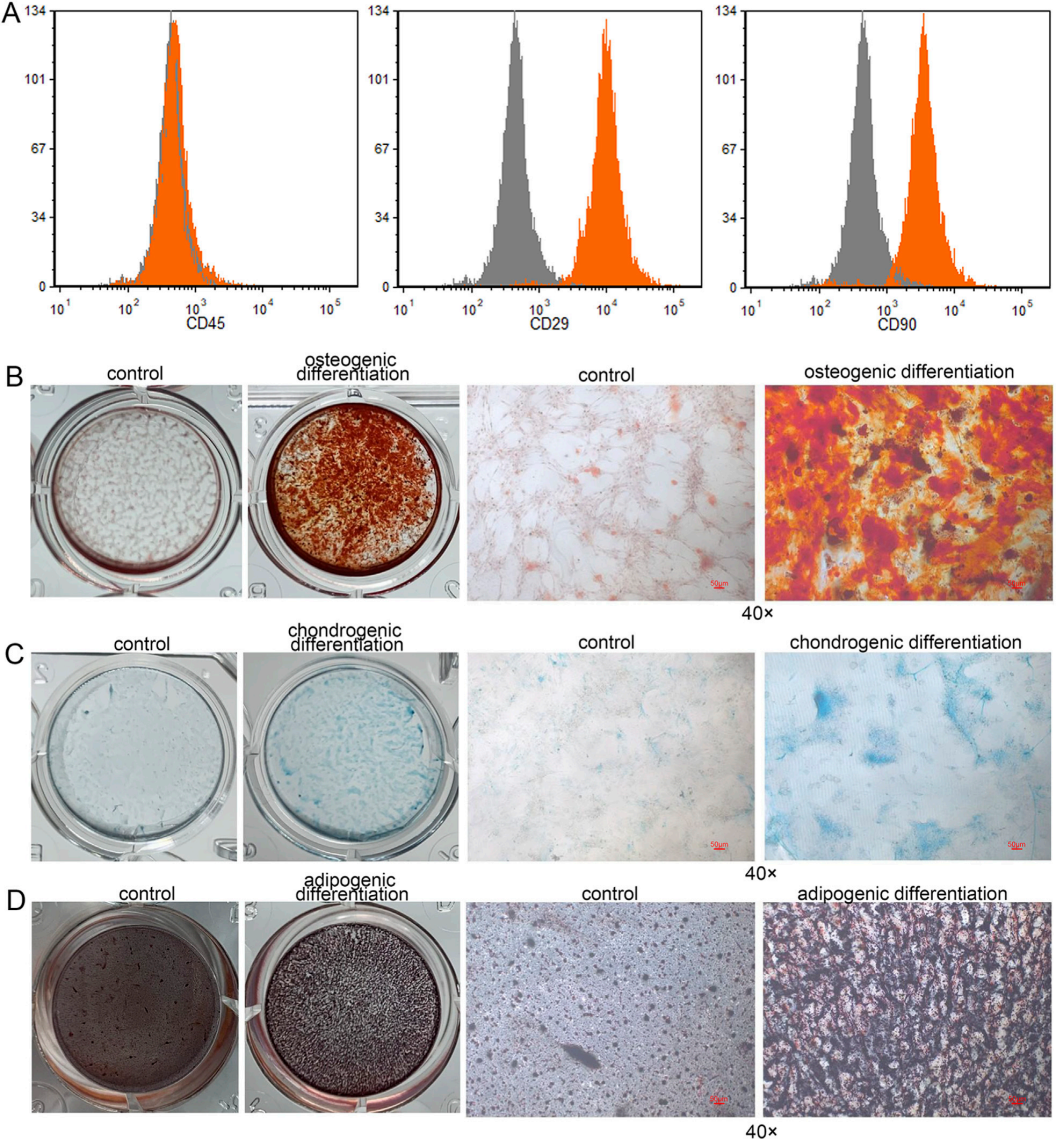
Identification and differentiative capacity of DPSCs. **(A)** Flow cytometry analysis was performed to characterize the isolated human dental pulp stem cells (DPSCs) by detecting the surface markers CD45, CD90, and CD29. **(B)** Osteogenic differentiation of human DPSCs. **(C)** Chondrogenic differentiation of human DPSCs. **(D)** Adipogenic differentiation of human DPSCs.

Flow cytometry analysis revealed that the isolated human DPSCs exhibited strong positive expression of CD90 and CD29, with expression rates of 90.16% ± 1.00% and 99.27% ± 0.11%, respectively. In contrast, the CD45 marker was negatively expressed (0.95% ± 0.09%) (Figure 1A). In this study, the multilineage differentiation capacity of the DPSCs was assessed, including osteogenic, chondrogenic, and adipogenic potential, verified by Alizarin Red (Figure 1B), Alcian Blue (Figure 1C), and Oil Red O staining (Figure 1D), respectively.

### 3.2 Effects of composite scaffolds on the expression of macrophage-related markers and pathway-related markers *in vitro*

**FIGURE 2:**
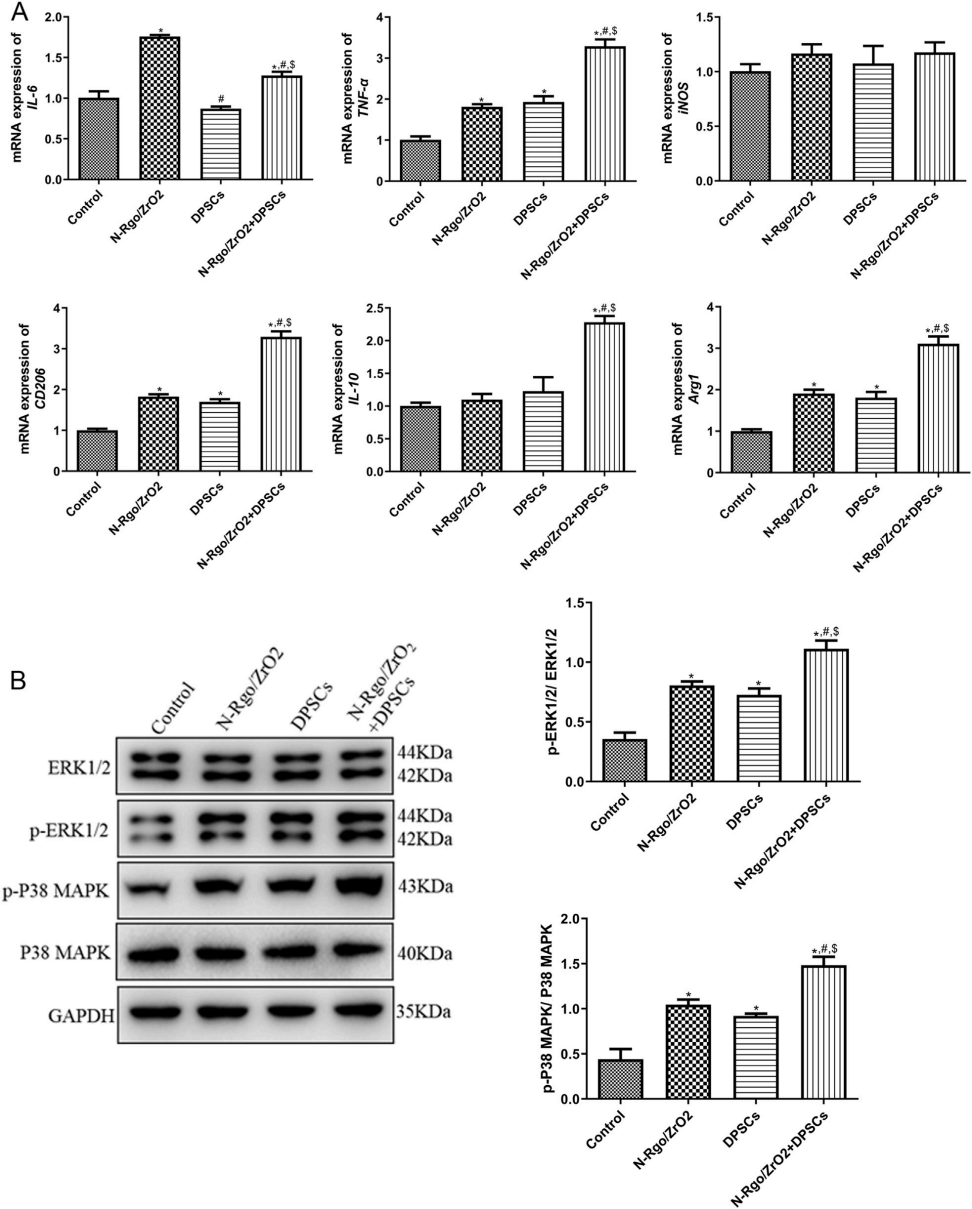
Effects of composite scaffolds on the expression of macrophage-related markers and pathway-related markers *in vitro*. The cells were divided into four experimental groups: control, N-Rgo/ZrO_2_, DPSCs, and N-Rgo/ZrO_2_+DPSCs group. **(A)** The mRNA expression of *IL-6, TNF-α, iNOS, CD206, IL-10* and *Arg1*. **(B)** Expression levels of p-ERK1/2 and p-P38 MAPK. N = 3. *P < 0.05 vs. control group; #*P* < 0.05 vs. N-Rgo/ZrO_2_ group; $*P* < 0.05 vs. DPSC group.

In the Thp-1 macrophages, the N-Rgo/ZrO_2_ group exhibited markedly increased mRNA levels of IL-6, TNF-α, CD206, and Arg1 compared with controls (P < 0.05), whereas iNOS and IL-10 expression remained unchanged (P > 0.05, Figure 2A). In the DPSCs-treated Thp-1 macrophages, the mRNA expression of TNF-α, CD206 and Arg1 was significantly upregulated, whereas IL-6, iNOS, and IL-10 exhibited no significant changes. Furthermore, the N-Rgo/ZrO_2_+DPSCs--treated Thp-1 macrophages demonstrated extremely significant upregulation in the mRNA expression of IL-6, TNF-α, CD206, IL-10, and Arg1, while iNOS mRNA levels remained unchanged. Compared with the N-Rgo/ZrO_2_ group, the DPSC group exhibited significantly downregulated IL-6 mRNA expression, with no notable differences in other genes. In contrast, the N-Rgo/ZrO_2_+DPSCs group showed extremely significant downregulation of IL-6 mRNA, significant upregulation of TNF-α, CD206, IL-10, and Arg1, and no change in iNOS expression. Compared with the DPSC group, the N-Rgo/ZrO_2_+DPSCs group displayed significantly increased mRNA expression of IL-6, TNF-α, CD206, IL-10, and Arg1, while iNOS remained unaffected (Figure 2A). Further investigations revealed that treatment with N-Rgo/ZrO_2_ or DPSCs alone significantly increased the protein expression levels of p-ERK1/2 and p-p38 MAPK, whereas N-Rgo/ZrO_2_+DPSCs induced a more pronounced upregulation of p-ERK1/2 and p-p38 MAPK compared with N-Rgo/ZrO_2_ or DPSC treatment alone (P < 0.05, Figure 2B).

### 3.3 Effects of composite scaffolds on rat jaw injury model *in vivo*

**FIGURE 3:**
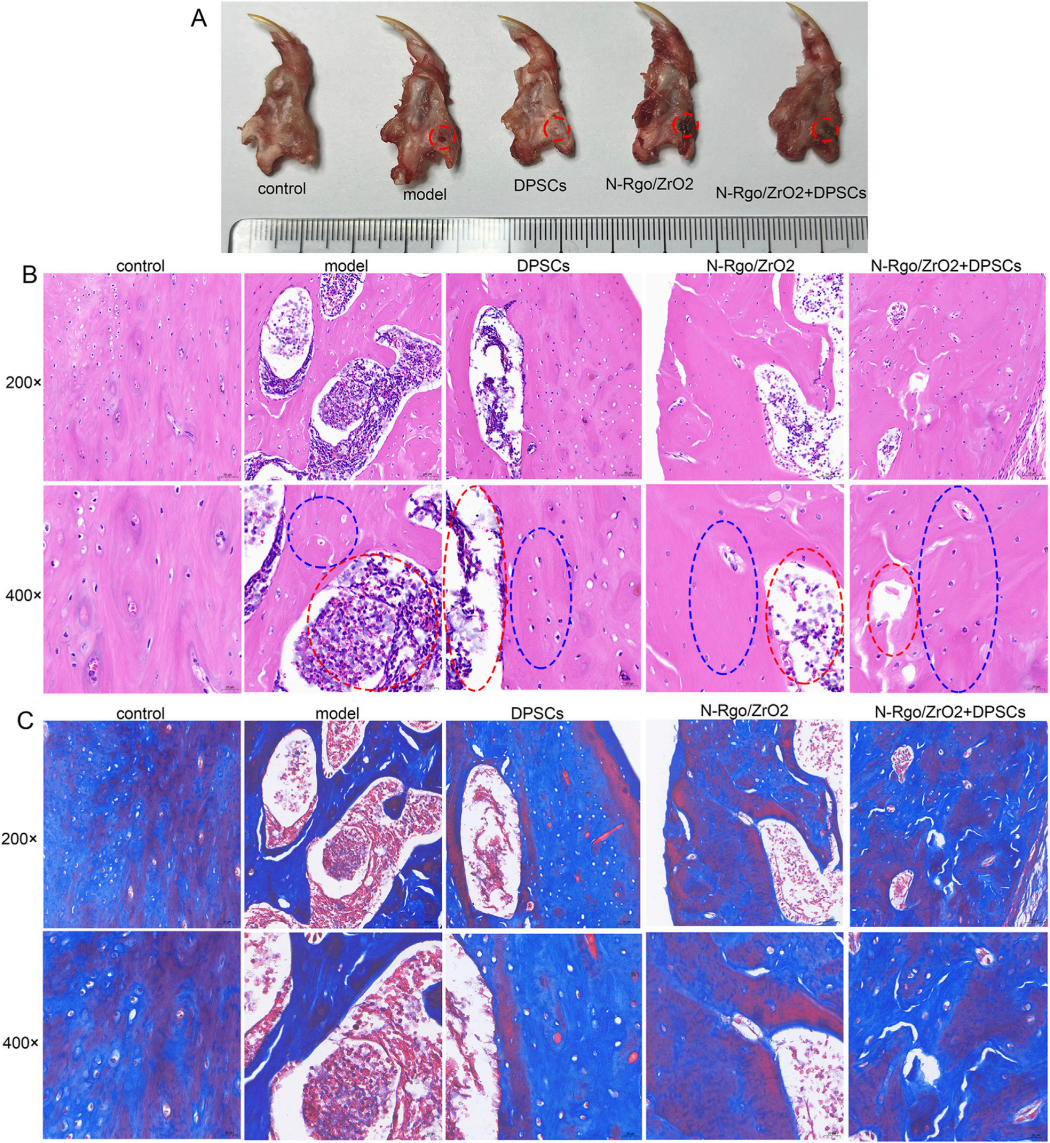
Effects of composite scaffolds on rat jaw injury model *in vivo*. **(A)** Thirty Wistar rats were randomly allocated into five groups (n = 6 per group): control (sham-operated, no treatment), model (disease-induced, untreated), DPSCs (disease model + dental pulp stem cell transplantation), N-RGO/ZrO_2_ (disease model + nanocomposite only), and N-RGO/ZrO_2_+DPSCs (disease model + combined treatment). The red circle indicates the location of the bone defect. **(B)** H&E staining was performed to evaluate pathological changes in the maxillary bone tissue. The red circle indicates the area of the bone defect and the blue circle indicates the newly formed bone region. **(C)** Masson staining was performed to evaluate pathological changes in maxillary bone tissue.

In this study, a rat bone defect model was constructed. After the rats were anesthetized, the left submandibular area was disinfected. An incision was made in the skin parallel to the inferior edge of the mandible, and the skin and subcutaneous tissue were incised sequentially. The muscle tissue and periosteum were dissected to expose the surface of the mandible, and a bone drill was used to create a circular full-thickness bone defect (Figure 3A). In addition, HE results showed no bone defect in the control rats. Compared with the control rats, the model rats exhibited structural disruption of trabecular bone, with reduced and thinned osseous and osteoid tissues, or even complete disappearance. The cortical bone became thinner, and the medullary cavity was relatively enlarged. The lining of osteoblasts along the bone surface decreased, whereas osteoclast activity increased, with surface erosion, indicating an altered matrix composition by marrow stromal cells (Figure 3B). Treatment with N-Rgo/ZrO_2_ or DPSCs alone partially ameliorated these pathological conditions, whereas cotreatment with N-Rgo/ZrO_2_ and DPSCs further improved the mandibular bone tissue destruction in the model rats (Figure 3B). Furthermore, Masson’s trichrome staining showed that, compared with the control group, the mandibular bone tissue of the model group rats exhibited significantly reduced blue-stained collagen fibers, disorganized arrangement of trabecular bone collagen matrix, increased red-stained noncollagenous components, widened marrow spaces, and thinning of trabeculae. In contrast to the model group, treatment with N-Rgo/ZrO_2_ or DPSCs alone increased collagen fiber density, partially restored parallel collagen fiber alignment, significantly reduced fibrotic degeneration, and moderately improved trabecular connectivity. In the N-Rgo/ZrO_2_+DPSCs cotreatment group, the collagen fiber density and tissue organization approached near-normal levels, with complete restoration of the trabecular network (Figure 3C).

### 3.4 Effects of composite scaffolds on the expression of macrophage-related markers *in vivo*

**FIGURE 4:**
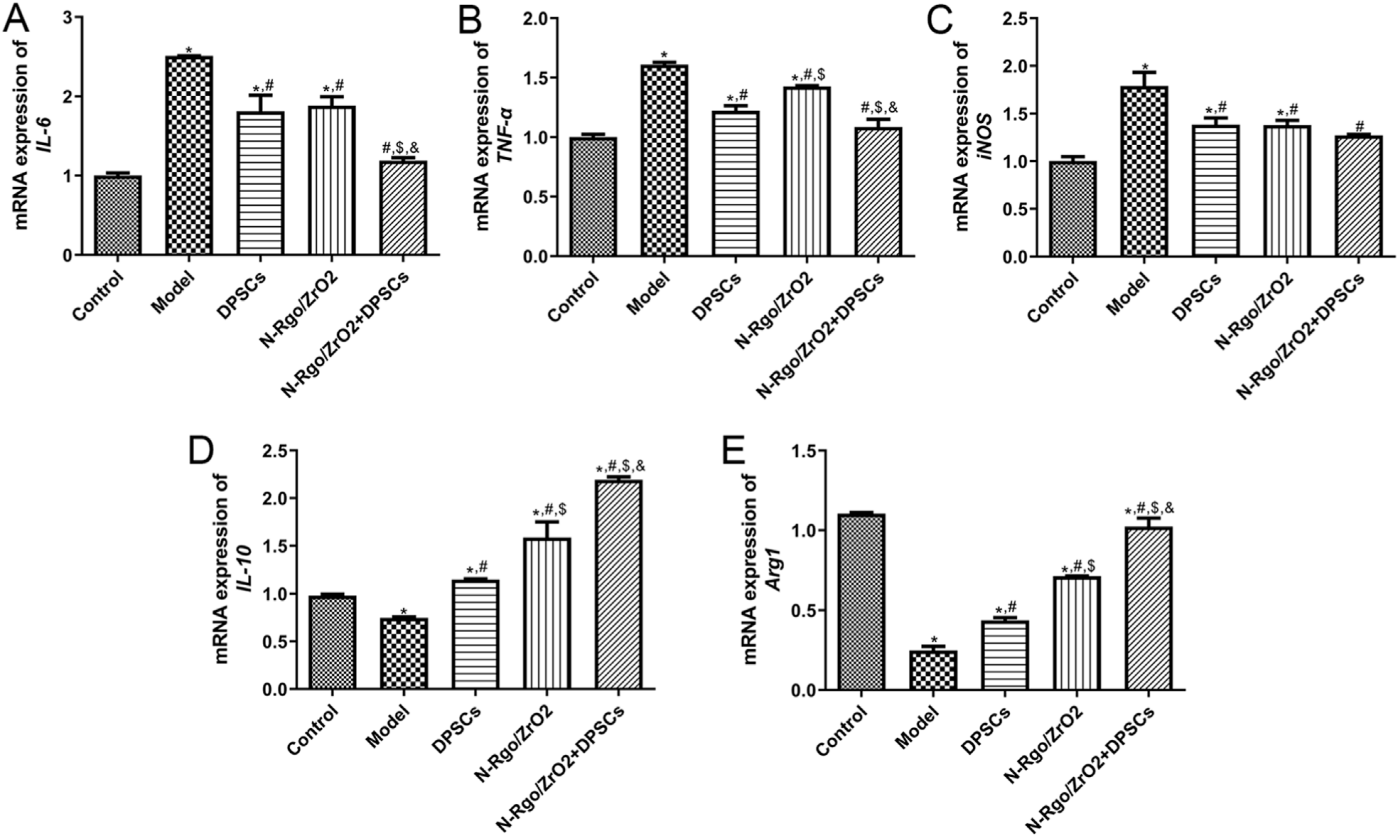
Effects of composite scaffolds on the expression of macrophage-related markers *in vivo*. Maxillofacial bone tissues were isolated from all five experimental groups of rats. **(A)** The mRNA expression of *IL-6*. **(B)** The mRNA expression of *TNF-α*. **(C)** The mRNA expression of *iNOS*. **(D)** The mRNA expression of *IL-10*. **(E)** mRNA expression of *Arg1*. N = 3. *P < 0.05 vs. control group; #*P* < 0.05 vs. model group; $*P* < 0.05 vs. DPSCs group; &*P* < 0.05 vs. N-RGO/ZrO_2_ group.

Compared with the control group, the model group exhibited notably increased mRNA expression of IL-6, TNF-α, and iNOS, while the mRNA expression of IL-10 and Arg1 was significantly downregulated. After DPSC treatment, the mRNA expression of IL-6, TNF-α, and iNOS was significantly downregulated (Figures 4A–C), whereas the mRNA expression of IL-10 and Arg1 was significantly upregulated (Figures 4D,E). Following N-Rgo/ZrO_2_ treatment, the mRNA expression of IL-6 was significantly downregulated, while TNF-α and iNOS showed a downward trend (Figures 4A–C), whereas the mRNA expression of IL-10 and Arg1 was upregulated (Figures 4D,E). Cotreatment with N-Rgo/ZrO_2_ and DPSCs led to a significant downregulation of IL-6, TNF-α, and iNOS mRNA expression and a significant upregulation of IL-10 and Arg1 mRNA expression (P < 0.05, Figures 4A–E).

### 3.5 Effects of composite scaffolds on the expression of osteogenic differentiation-related markers and pathway-related markers *in vivo*

**FIGURE 5:**
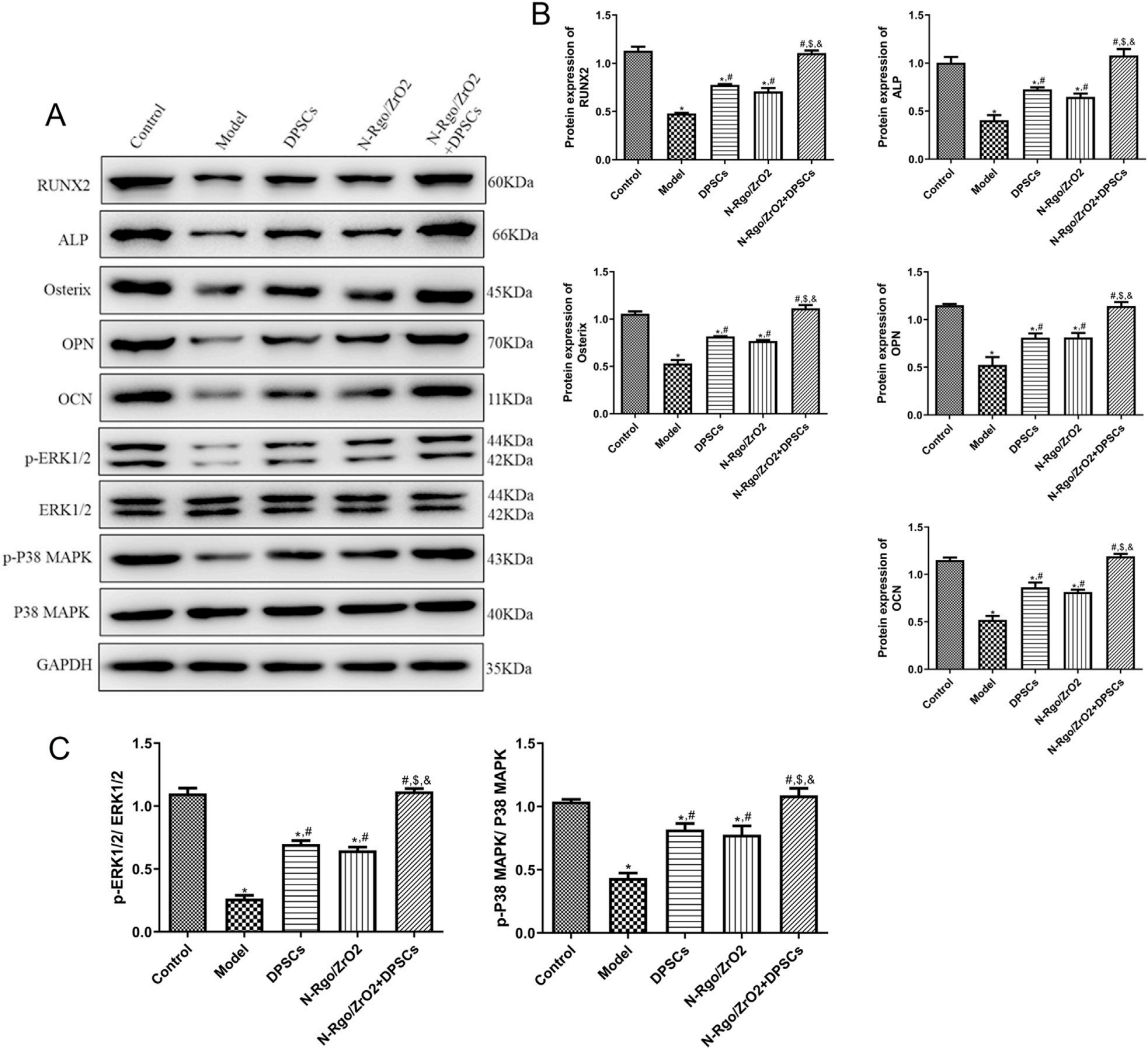
Effects of composite scaffolds on the expression of osteogenic differentiation-related markers and pathway-related markers *in vivo*, Maxillofacial bone tissues were isolated from all five experimental groups of rats. **(A,B)** The protein expression of RUNX2, ALP, Osterix, OPN, and OCN in maxillofacial bone tissues. **(C)** The protein expression of p-ERK1/2 and p-P38 MAPK in maxillofacial bone tissues. N = 3. *P < 0.05 vs. control group; #*P* < 0.05 vs. model group; $*P* < 0.05 vs. DPSCs group; &*P* < 0.05 vs. N-RGO/ZrO_2_ group.

The model group exhibited significantly reduced expression of RUNX2, ALP, Osterix, OPN, OCN, p-ERK1/2, and p-P38 MAPK relative to the controls (P < 0.05, Figures 5A–C). Treatment with N-Rgo/ZrO_2_ or DPSCs alone upregulated the expression levels of RUNX2, ALP, Osterix, OPN, OCN, p-ERK1/2, and p-P38 MAPK compared with the model group (P < 0.05, Figures 5A–C). Compared with treatment with N-Rgo/ZrO_2_ or DPSCs alone, the combined treatment with ZrO_2_/DPSCs further increased the expression levels of RUNX2, ALP, Osterix, OPN, OCN, p-ERK1/2, and p-P38 MAPK (P < 0.05, Figures 5A–C).

## 4 Discussion

Maxillofacial bone regeneration requires simultaneous structural and immunological regulation; however, current DPSC-based therapies often fail to address the dysregulation of the inflammatory microenvironment. In this study, an N-Rgo/ZrO2 scaffold was engineered that synergizes with DPSCs through dual biointerface interactions. *In vitro*, the composite activated ERK/p38 MAPK pathways, driving RUNX2-mediated osteogenesis and M2 macrophage polarization through TNF-α/IL-10 rebalancing. In rats with bone defects, this synergy enhanced mandibular regeneration, increased collagen deposition, and improved osteogenic marker network activation compared with single treatments. The synergistic bone regenerative potential of DPSCs combined with N-Rgo/ZrO_2_ composite scaffolds was elucidated, highlighting their dual roles in immunomodulation and osteogenic differentiation. The findings provide compelling evidence that this combinatorial strategy significantly enhances mandibular bone repair in a rat bone defect model, surpassing the effects of DPSCs or N-Rgo/ZrO_2_ scaffolds alone.

DPSCs and bone marrow mesenchymal stem cells (BMSCs) are important mesenchymal stem cell sources for bone regeneration; however, they exhibit distinct characteristics that influence their application potential, particularly in maxillofacial bone repair (Fujii et al., 2023; Zhang et al., 2024). Originating from neural crest cells, DPSCs possess a unique ontogenetic relationship with craniofacial tissues, endowing them with inherent specificity for maxillofacial bone regeneration (Yasui et al., 2017), which is not matched by BMSCs derived from mesoderm. In terms of accessibility, DPSCs can be isolated from extracted teeth through minimally invasive procedures with low donor-site morbidity (Fujii et al., 2023), whereas BMSCs require invasive bone marrow aspiration, which limits their clinical feasibility. Functionally, DPSCs demonstrate superior immunomodulatory capacity (Fu et al., 2022), specifically polarizing macrophages toward the pro-regenerative M2 phenotype compared with the relatively broad and less targeted immune regulation of BMSCs. Although both exhibit osteogenic potential, DPSCs show enhanced mineralization efficiency in craniofacial microenvironments, with robust expression of bone-related markers under inductive conditions (Lee et al., 2019). These differences highlight DPSCs as a more promising candidate for maxillofacial bone repair, aligning with the need for effective strategies in trauma, osteoporosis, or post-resection rehabilitation scenarios. Thus, exploring DPSCs in combination with functional biomaterials, such as N-Rgo/ZrO_2_, has significant potential to address the clinical challenges of maxillofacial bone regeneration.In this study, the observed upregulation of M2 macrophage markers (CD206, Arg1, and IL-10) and suppression of IL-6 in N-Rgo/ZrO_2_+DPSCs-treated Thp-1 macrophages corroborates the emerging evidence that DPSCs exert potent immunomodulatory effects. Similar to the results of this investigation, a previous study by Sun et al. (Sun et al., 2021) found that MSCs seeded on GO-modified silk fibroin (SF)/nanohydroxyapatite (nHA) scaffolds polarize macrophages toward an M2 phenotype and accelerate bone regeneration, thereby enhancing calvarial defect repair in rats. However, in this study, this paradigm was advanced by demonstrating that N-doping further amplifies DPSC immunomodulation, likely owing to improved scaffold conductivity and protein adsorption (Zhang et al., 2023). Compared with other stem cell types used in bone regeneration, DPSCs appear to be particularly effective for modulating macrophage responses. Although adipose-derived stem cells (ADSCs) have shown similar potential (Kadk et al., 2024), DPSCs may offer advantages in craniofacial applications because of their neural crest origin (Janebodin et al., 2011). The zirconia component of the scaffold may further contribute to immunomodulation because recent studies have demonstrated that ZrO_2_ nanoparticles can scavenge reactive oxygen species in inflammatory environments (An et al., 2022). This synergistic immunomodulation, arising from the N-Rgo-enhanced DPSC paracrine activity and ZrO_2_-mediated oxidative stress mitigation, establishes a self-reinforcing regenerative loop where M2 macrophage-derived TNF-α further amplifies MAPK-driven osteogenesis, thereby transcending traditional biomaterial approaches that passively rely on either cellular or material components alone.

The MAPK pathway can be divided into several main branches, each comprising different MAPK proteins, including the ERK, JNK, and p38 MAPK pathways (Huang et al., 2024; Ma and Nicolet, 2023). The ERK pathway primarily responds to growth factors and some other stimuli and is typically associated with cell proliferation and differentiation processes (Park, 2023; Wen et al., 2022). It has been reported that the MAPK and ERK pathways play critical roles in osteogenic differentiation (Zeng et al., 2024). The robust activation of the p-ERK1/2 and p-p38 MAPK pathways in this study provides mechanistic insight into the enhanced osteogenesis observed with N-Rgo/ZrO2+DPSCs. These findings are consistent with those of a recent study showing that ERK1/2 activation is crucial for DPSC osteogenic differentiation on bioactive glass scaffolds (Yang et al., 2024). However, this study uniquely demonstrated that the N-Rgo/ZrO_2_ composite amplifies MAPK phosphorylation beyond the effects of DPSCs alone, suggesting a biomaterial-mediated priming effect. This aligns with emerging evidence that graphene-based materials can activate mechanosensitive ion channels (e.g., Piezo1) in stem cells, triggering downstream MAPK cascades (Kim et al., 2015). In addition, RUNX2, a core transcription factor regulating osteoblast differentiation, is highly expressed during the differentiation of mesenchymal stem cells into osteogenic precursor cells (Komori, 2022). Osterix, a downstream transcription factor of RUNX2, specifically regulates the terminal differentiation of osteoblast OCN as a terminal differentiation marker of mature osteoblasts, reflecting the mineralization of bone matrix (Zeng et al., 2024; Liu et al., 2020). The upregulation of RUNX2, Osterix, and OCN in the *in vivo* model in this study compares favorably with recent clinical trials using DPSC-seeded 3D-printed multiphase scaffolds for periodontal regeneration (Lee et al., 2014). The composite achieved superior collagen organization, probably because of the mechanical stability of zirconia under load, which is a significant advantage over softer polymeric scaffolds that may collapse in mandibular defects (Chin et al., 2021). The scaffold alone exhibited limited osteoinductive properties, emphasizing the need for DPSCs for functional bone repair. This aligns with prior reports that stem-cell-seeded scaffolds outperform acellular biomaterials in critical-sized defects (Singh et al., 2022). The significant elevation of ALP, OPN, and OCN—important markers of late osteogenic differentiation—further confirms the maturation of regenerated bone in the cotreatment group. ALP activity is the most widely recognized marker of osteoblast activity and is a typical protein product of osteoblast phenotype and differentiation (Fisher and Schöck, 2022). OPN, a major noncollagenous bone matrix protein secreted by osteoblasts and osteoclasts, potently enhances both osteoclast differentiation and mature osteoclast resorptive activity (Lin et al., 2023). Furthermore, it was found that OCN, expressed during terminal osteoblast differentiation, regulates bone mineralization and calcium homeostasis through Ca^2+^ binding (Ching et al., 2017). These results suggest that the proposed approach is particularly suitable for load-bearing craniofacial applications. Collectively, these results may delineate a N-Rgo/ZrO_2_-mediated mechanotransduction axis wherein coordinated ERK/p38 MAPK activation synergizes with DPSC-derived osteogenic programming to drive RUNX2/OSX-dependent bone matrix maturation, while the load-bearing stability of zirconia can ensure structural integrity during mandibular defect remodeling—a dual functionality critical for translating scaffold-based therapies into functional craniofacial reconstruction.

Compared with pure GO scaffolds, the prepared N-Rgo/ZrO_2_ composite exhibits distinct advantages in bone regeneration, primarily attributed to the synergistic effects of nitrogen doping and zirconia integration. First, nitrogen doping in N-Rgo introduces additional active sites and enhances surface charge distribution, which improves protein adsorption and cellular adhesion-critical factors for osteogenic differentiation and scaffold integration in hDPSCs (Achôa et al., 2023; Taşdemir et al., 2021). In contrast, GO lacks these nitrogen-mediated bioactivity enhancements (Khosalim et al., 2022). Second, the incorporation of ZrO_2_ into the composite provides mechanical stability and osteoconductivity, which are absent in GO scaffolds. The high compressive strength of ZrO_2_ is particularly advantageous for load-bearing maxillofacial applications, whereas GO alone may lack the structural integrity required for such defects (Daud et al., 2025; Khaledi et al., 2018). Third, N-Rgo/ZrO_2_ exhibits superior electrical conductivity compared with GO, which facilitates cellular communication and MAPK pathway activation (Bellet et al., 2021; Farjaminejad et al., 2024), as demonstrated by the robust upregulation of p-ERK1/2 and p-p38 in this study. This conductivity-driven osteogenic signaling is less pronounced in GO scaffolds. Finally, the unique nanotopography of N-Rgo/ZrO_2_ mimics the bone ECM more effectively than GO, promoting DPSC attachment and proliferation (Yao et al., 2021). While GO has been explored in bone tissue engineering, its limitations in bioactivity and mechanical support underscore the need for such composite strategies as N-Rgo/ZrO_2_ to achieve synergistic immunomodulation and osteogenesis (Gostaviceanu et al., 2024). In contrast to the standalone ZrO_2_ scaffolds, which lack inherent osteoinductivity, the developed composite achieved significantly higher ALP activity and mineralization through DPSC incorporation. Relative to polymer-based systems (e.g., PCL) (Terranova et al., 2021), the scaffold in this study induced earlier and more-robust osteogenic marker expression, potentially because of the enhanced charge transfer at the cell–material interface. Collectively, these differences highlight that N-Rgo/ZrO_2_ outperforms pure GO in bone regeneration by combining enhanced surface bioactivity, electrical conductivity, targeted immunomodulation, and mechanical stability, making it a more effective scaffold for synergizing with DPSCs in maxillofacial bone repair.

However, this study had some limitations. First, the *in vivo* experiments revealed changes in cytokine expression within bone tissue, but the cellular origin of these cytokines should be measured further. Second, histological analyses of bone tissue recovery *in vivo* need to be quantified using micro-CT, and information on macrophages within the tissues at earlier time points also should be further assessed. Third, the conclusions need to be validated using MAPK inhibitors. In addition, while the rat bone defect model employed addresses a critical clinical need, translation faces several challenges. First, autologous DPSC isolation is more invasive than ADSC harvesting (Rajasingh et al., 2021), which has motivated research into allogeneic or iPSC-derived alternatives. Second, although zirconia exhibits exceptional compressive strength, its fatigue resistance under cyclic loading requires evaluation. Based on the findings, it is proposed that single-cell RNA sequencing should be employed to characterize DPSC heterogeneity within the scaffold environment, alongside large-animal studies to assess the healing of critical-sized defects.

In conclusion, this study established the N-Rgo/ZrO_2_+DPSCs composite as a multifaceted strategy for maxillofacial bone regeneration, operating through synergistic immunomodulatory and osteogenic mechanisms. The composite scaffolds may exert their protective effects through the coordinated activation of the ERK1/2 and p38 MAPK signaling pathways, driving both M2 macrophage polarization and osteogenic differentiation through RUNX2/OSX transcriptional activation. The ability of the scaffold to amplify DPSC paracrine signaling and differentiation represents a significant advance over previous monotherapies. Future work should focus on bridging the gap between preclinical models and human applications, particularly in complex clinical scenarios, such as aged or diabetic bone.

## Data Availability

The original contributions presented in the study are included in the article/supplementary material, further inquiries can be directed to the corresponding author.
